# The RING-Type E3 Ubiquitin Ligase Gene *GhDIRP1* Negatively Regulates *Verticillium dahliae* Resistance in Cotton (*Gossypium hirsutum*)

**DOI:** 10.3390/plants13152047

**Published:** 2024-07-25

**Authors:** Fenglin Miao, Wei Chen, Yunlei Zhao, Pei Zhao, Xiaohui Sang, Jianhua Lu, Hongmei Wang

**Affiliations:** 1 State Key Laboratory of Cotton Bio-Breeding and Integrated Utilization, Institute of Cotton Research, Chinese Academy of Agricultural Sciences, Anyang 455000, China; miaofenglin2021@163.com (F.M.); zhaoyunlei@caas.cn (Y.Z.); zhaopei@caas.cn (P.Z.); sangxiaohui@caas.cn (X.S.); 2 Zhengzhou Research Base, State Key Laboratory of Cotton Bio-Breeding and Integrated Utilization, Zhengzhou University, Zhengzhou 450000, China

**Keywords:** cotton, verticillium wilt, E3 ubiquitin ligase, *GhDIRP1*, disease resistance

## Abstract

Cotton is one of the world’s most important economic crops. Verticillium wilt is a devastating cotton disease caused by *Verticillium dahliae*, significantly impacting cotton yield and quality. E3 ubiquitin ligases are essential components of the ubiquitin-mediated 26S proteasome system, responsible for recognizing ubiquitinated target proteins and promoting their degradation, which play a crucial regulatory role in plant immune responses. In this study, on the basis of the confirmation of differential expression of *GhDIRP1*, a RING-type E3 ubiquitin ligase encoding gene, in two cotton varieties resistant (Zhongzhimian 2) or susceptible (Jimian 11) to *V. dahliae*, we demonstrated that *GhDIRP1* is a negative regulator of *V. dahliae* resistance because silencing *GhDIRP1* in cotton and heterogeneously overexpressing the gene in Arabidopsis enhanced and compromised resistance to *V. dahliae*, respectively. The *GhDIRP1*-mediated immune response seemed to be realized through multiple physiological pathways, including hormone signaling, reactive oxygen species, and lignin biosynthesis. Based on the sequences of *GhDIRP1* isolated from Zhongzhimian 2 and Jimian 11, we found that *GhDIRP1* had identical coding but different promoter sequences in the two varieties, with the promoter of Zhongzhimian 2 being more active than that of Jimian 11 because the former drove a stronger expression of GUS and LUC reporter genes. The results link the ubiquitination pathway to multiple physiological pathways acting in the cotton immune response and provide a candidate gene for breeding cotton varieties resistant to *V. dahliae*.

## 1. Introduction

Cotton is one of the world’s major economic crops, widely grown globally, and its products have wide applications in various fields [1]. Since the 1980s, cotton production in China has continually expanded, making it the world’s largest cotton-producing country and a vital contributor to its economic development [2]. Upland cotton (*G. hirsutum* L.) and Pima cotton (*G. barbadense* L.) are the two widely cultivated tetraploid cotton species, accounting for 97% and 3% of the world’s cotton production area, respectively [3]. While Upland cotton has a higher yield potential than Pima cotton, it has the disadvantage of poor resistance to Verticillium wilt (VW) and lower fiber quality. By contrast, Pima cotton exhibits better fiber quality and higher resistance to VW but has lower yield and is less adaptable to various ecological conditions [4].

Vertcillium wilt (VW), a soil-borne fungal disease, leads to serious yield loss in many cotton-producing countries [5,6]. VW is caused by the soil-borne fungal pathogen *V. dahliae*, which infects cotton through roots, causing systemic infection throughout the entire growth stage of plants, from seedlings to maturity. Symptoms of VW in cotton seedlings include wilting and softening of the leaf edges, resembling dehydration, as well as browning and wilting of the leaves at later developmental stages [7]. The pathogen proliferates extensively within the vascular system, hindering the transport of sugars and water, leading to plant wilting. The degree of infection of vascular pathogens in host plants depends on two factors: the pathogen must have the ability to penetrate into the host vascular bundles, and once inside, a small amount of mycelium can form a complete systemic infection [8]. After invading, the VW pathogen occludes the vascular bundles, impairing the transportation of water and nutrients. Combined with the vigorous transpiration and respiration in the aboveground parts, this water imbalance leads to plant wilting. The more severe the *V. dahliae* infection, the more vascular bundles are obstructed by fungal mycelium [9].

The 26S proteasome system mediated by ubiquitin (Ub) is the main pathway for protein degradation in plant cells, participating in the degradation of many intracellular proteins [10,11]. The Ub system consists of five basic components: ubiquitin-activating enzyme (E1s), ubiquitin-conjugating enzyme (E2s), ubiquitin-ligating enzyme (E3s), ubiquitin, and the 26S proteasome [12,13]. The typical Ub-mediated protein degradation process involves initially activating the highly conserved Ub small-molecule protein by E1s and transferring it to E2s. Ub is then transferred to E3s, which, in turn, marks the target protein with Ub, forming mono-Ub or multi-Ub tagged proteins. These tagged proteins are subsequently sent for degradation through the 26S proteasome system [14].

A study showed that the plant Ub-proteasome system is associated with immune responses. In cotton, the U-box type E3 Ub ligase *GhPUB17* negatively regulates cotton resistance to *V. dahliae*. The antifungal protein GhCyP3 can interact with GhPUB17, inhibiting its ligase activity and playing an important role in the cotton-*V. dahliae* interaction [15,16,17]. Patatin-like proteins (PLPs) have non-specific acyl hydrolase activity and can hydrolyze membrane lipids into fatty acids and phospholipids. The expression of the cotton patatin-like protein encoding gene *GhPLP2* is induced by *V. dahliae*, and phytohormones jasmonic acid (JA) and ethylene (ETH). *GhPLP2* is involved in cotton resistance to *V. dahliae* by maintaining the fatty acid metabolic pool used for JA biosynthesis and activating the JA signaling pathway [18]. In rice, the RING-type ubiquitin ligase protein APIP10 interacts with two rice transcription factors, OsVOZ1 and OsVOZ2, promoting their degradation through the 26S proteasome pathway and negatively regulating basal defense, playing a positive role in rice immunity [19,20]. Rice ubiquitin-conjugating enzyme OsUBC26 has the effect of resisting rice blast fungus. *OsUBC26* expression is induced by rice blast fungus infection and jasmonic acid methyl ester treatment. OsUBC26 plays an important role in rice disease resistance by regulating WRKY45 expression and interacting with the E3 ligase APIP6 [21,22]. In wheat, small-secreted proteins (ZtSSPs) play a critical role in the successful colonization of host tissues. ZtSSP2 interacts with wheat E3 ubiquitin ligase (TaE3UBQ), which plays a vital role in plant immunity [20]. In Arabidopsis, receptor-like cytoplasmic kinase BIK1 acts as a signaling hub in plant immunity. The stability of BIK1 is maintained by a regulatory module, in which CPK28 modulates BIK1 conversion by regulating the activities of two E3 ligases. CPK undergoes ubiquitination and 26S proteasome-mediated degradation, and flagellin treatment enhances this degradation. ATL31 and ATL6 interact specifically with CPK28 on the plasma membrane, directly ubiquitinating CPK28, leading to its proteasomal degradation [23,24,25]. Brassinosteroids (BRs) regulate plant growth, development, and stress responses by activating the core transcription factor BES1, whose degradation occurs through the proteasome and autophagy pathways. In Arabidopsis, the E3 ubiquitin ligase BAF1 interacts with BES1, mediating its ubiquitination and degradation [26]. VDAL protein in Arabidopsis competes with the transcription factor MYB6 for binding to E3 ligases PUB25/PUB26. Binding of VDAL protein to PUB prevents MYB6 ubiquitination and degradation, promoting resistance to VW [17]. Additionally, the ubiquitin/26S proteasome pathway is involved in the regulation of fiber development. The E3 ubiquitin ligase GhHUB2 interacts with the cotton fiber development transcription repressor GhKNL1 protein, ubiquitinating and degrading GhKNL1 through the UPS pathway, thus participating in the molecular regulation of cotton fiber development [27,28]. The aforementioned studies mainly focus on the interaction between E3 ubiquitin ligases and target proteins and their roles in immune function. However, the molecular mechanisms underlying the interaction between E3 ubiquitin ligases and their substrate proteins and their involvement in immune signal regulation remain elusive.

In our previous study, *Gh_D03G138200*, a cotton homolog of the Arabidopsis E3 ligase gene *AtRDUF1*, was identified to be responsive to *V. dahliae* infection based on RNA-Seq analysis [29]. In this study, we experimentally characterized the function of *Gh_D03G138200* or *GhDIRP1* in response to *V. dahliae* infection. We analyzed the expression patterns of *Gh_D03G138200* upon *V. dahliae* infection and hormone treatment in resistant and susceptible cotton genotypes, investigated the function of the gene in VW resistance using VIGS in cotton and overexpression in Arabidopsis. We cloned the gene from the *V. dahlia*-resistant cotton variety Zhongzhimian 2 (ZZM2) and the susceptible variety Jimian 11 (JM11) and compared their structure and protein properties. The ubiquitination activity of Gh_D03G138200 was verified by an in vitro experiment, and the active sites of the protein were determined. The results of this study shed light on the molecular mechanisms of the ubiquitin pathway in cotton resistance against *V. dahliae*.

## 2. Results

### 2.1. GhDIRP1 Expression Changes Induced by Verticillium dahliae Infection and Hormone Treatment

**Figure 1:**
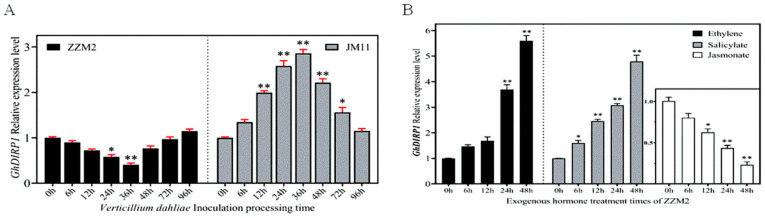
The expression profile of *GhDIRP1* in response to *V. dahliae* infection and hormone treatment. (**A**) qPCR quantification of the expression level of *GhDIRP1* after infection of *V. dahliae* in ZZM2 and JM11. (**B**) qPCR quantification of the expression level of *GhDIRP1* in response to hormone-treatment in ZZM2. * and ** indicate statistically significance at *p* < 0.05 and *p* < 0.01, respectively.

Previously, we found *Gh_D03G138200* or *GhDIRP1*, a homolog of the Arabidopsis E3 ligase gene *AtRDUF1*, is responsive to *V. dahliae* infection [29]. Here, we further investigated the expression profiles of *GhDIRP1* upon *V. dahliae* infection in cotton varieties resistant (ZZM2) or susceptible (JM11) to *V. dahliae*. We found that, in JM11, the expression level of *GhDIRP1* increased gradually upon *V. dahliae* infection, peaked at 36 h after infection (hai), and then gradually decreased to reach its normal level at ~96 hai. In contrast, the expression level of *GhDIRP1* in the resistant variety ZZM2 was gradually decreased upon *V. dahliae* infection, reached the lowest at 36 hai, and then increased to the normal level at ~48 hai (Figure 1A). In response to the treatment of various hormones, the expression level of *GhDIRP1* in ZZM2 was induced by ethylene and salicylate but repressed by Jasmonate (Figure 1B), suggesting that *GhDIRP1* may be involved in the signaling pathways of all these hormones.

### 2.2. Silencing GhDIRP1 Enhances Cotton Resistance to Verticillium Wilt

**Figure 2:**
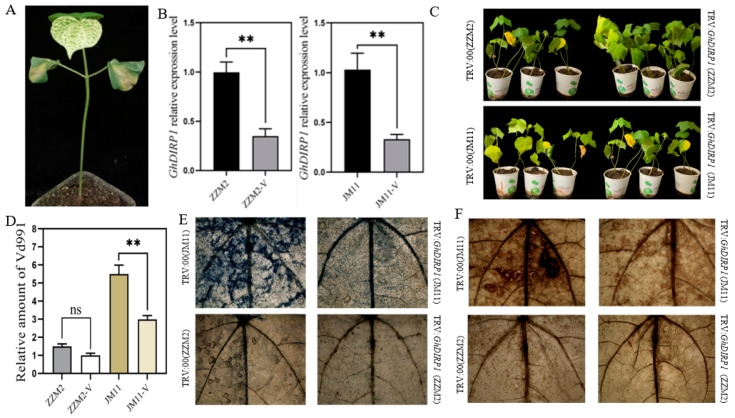
*GhDIRP1* is a negative regulator of resistance to *V. dahliae*. (**A**) The positive control of VIGS in which the cotton PDS gene was silenced to produce the albino phenotype. (**B**) qPCR results showing down-regulation of *GhDIRP1* in the VIGS plants. (**C**) Comparison of disease phenotype in *GhDIRP1*-silenced (TRV:*GhDIRP1*) and -unsilenced (TRV:00) plants after *V. dahliae* infection. (**D**) Quantification of the content of pathogen in the *GhDIRP1*-silenced (ZZM2-V and JM11-V) and -unsilenced (ZZM2 and JM11) plants. (**E**) Trypan staining of leaves from the *GhDIRP1*-silenced and -unsilenced plants infected by *V. dahliae*. (**F**) DAB-stained leaves from the *GhDIRP1*-silenced and -unsilenced plants infected by *V. dahliae*. ** indicates statistical difference at *p* < 0.01. ns: no significant difference.

The virus-induced gene silencing (VIGS) approach was used to down-regulate GhDIRP1 in both ZZM2 and JM11 and study the function of GhDIRP1 in cotton’s resistance to *V. dahliae* (Figure 2). Ten days after Agrobacterium infiltration, GhDIRP1 transcription was significantly inhibited, with its expression level reduced by 60% in the silenced plants of both varieties (Figure 2B). The GhDIRP1-silenced plants were infected by *V. dahliae*. Twenty days after infection, the leaves of TRV: GhDIRP1 plants of both ZZM2 and JM11 exhibited weaker symptoms, such as less wilting and discoloring, compared to the infected TRV:00 plants (Figure 2C). To accurately characterize the disease status of the plants, we compared the relative content of *V. dahliae* Vd991 in the roots of GhDIRP1-silenced and -unsilenced plants. The results showed that in ZZM2, the content of Vd991 did not change significantly before and after GhDIRP1 silencing, while in JM11, GhDIRP1 silencing significantly reduced the presence of Vd991 in the roots compared to the TRV:00 control plants (Figure 2D). To assess the cellular state of the plants after *V. dahliae* infection, leaves of the GhDIRP1-silenced and -unsilenced cotton seedlings were soaked in trypan blue dye. In the variety (JM11) susceptible to *V. dahliae*, compared to the GhDIRP1-unsilenced leaves, GhDIRP1-silenced (TRV:GhDIRP1) plants exhibited smaller and weaker blue areas around leaf veins (Figure 2E), indicating that GhDIRP1-silenced plants had less dead cells. Consistently, the accumulation of reactive oxygen species (ROS) in cotton leaves from GhDIRP1-silenced plants (TRV: GhDIRP1) was reduced. In the variety (ZZM2) resistant to *V. dahliae*, the levels of dead cells and ROS were not significantly different between the GhDIRP1-silenced and -unsilenced plants (Figure 2F). Together, these results imply that silencing GhDIRP1 enhanced cotton resistance to *V. dahliae* and that GhDIRP1 is a negative regulator of the resistance.

### 2.3. Silencing GhDIRP1 Increases SOD, POD, and NO Contents upon Verticillium dahliae Infection

The role of *GhDIRP1* in resistance to *V. dahliae* was further studied by measuring the content of H_2_O_2_, NO, SOD, and POD in the *GhDIRP1*-silenced and -unsilenced plants infected by *V. dahliae*. In both ZZM2 and JM11, after inoculation with *V. dahliae*, *GhDIRP1*-silenced plants exhibited reduced H_2_O_2_ and enhanced NO, SOD, and POD, and the content of chlorophyll and total protein increased (Supplementary Figure S1A–F). Between the two varieties, however, in both the *GhDIRP1*-silenced and -unsilenced plants, ZZM2 showed higher levels of NO, SOD, and POD than JM11.

### 2.4. Silencing GhDIRP1 Induces Expression Changes of the Genes Related to Lignin Biosynthesis and Hormone Signaling

In order to further elucidate the effect of *GhDIRP1* on resistance to *V. dahliae* in cotton, the expression levels of known disease resistance genes, including genes encoding the enzymes catalyzing the biosynthesis of lignin (*POD*, *PPO*, and *PAL*) and involving the JA and SA signaling pathways (*NPR1*, *PR1*, *PR3*, *PR5*, *LOX1*, *JAZ1*, *HIN1*), were compared in *GhDIRP1*-silenced (TRV: *GhDIRP1*) and -unsilenced (TRV:00) ZZM2 and JM11 plants before and after inoculation with *V. dahliae*. As is shown in Supplementary Figure S1, the expression level of *POD*, *PPO*, and *PAL* was induced by infection of *V. dahliae* at about 6–12 hai in TRV: *GhDIRP1* plants of both ZZM2 and JM11. The induced expression level reached peak at 12–24 hai in both varieties and then started to decrease. For the marker genes of the JA and SA signaling pathways, the expression level of all genes was upregulated in *GhDIRP1*-silenced plants comparing with -unsilenced plants; however, *NPR1* and *PR5* had the highest upregulated expression at 12 hai, *PR1* at 24–48 hai, *PR3* at 24 hai, *LOX1* and *HINT1* at 6–12 hai, and *JAZ1* at 6 hai in ZZM2 (Supplementary Figure S2A). In JM11, *NPR1* had the highest upregulated expression at 12 hai, *PR1* at 24 hai, *PR3* at 6 hai, *PR5* at 12 hai, *LOX1* increased the most during 24–48 hai, *JAZ1* and *HINT1* increased gradually from 12 to 48 hai (Supplementary Figure S2B). Overall, silencing *GhDIRP1* induces expression changes of the genes of lignin biosynthesis and hormone signaling.

### 2.5. Overexpressing GhDIRRP1 in Arabidopsis Compromises Resistance to Verticillium Wilt

**Figure 3:**
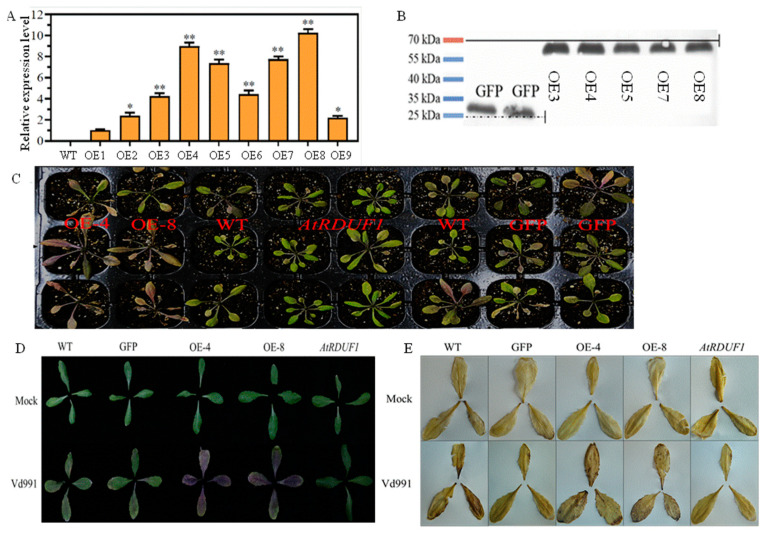
Overexpressing *GhDIRP1* in Arabidopsis compromises resistance to *V. dahliae*. (**A**) qRT-PCR results of the *GhDIRP1* transgene in different transgenic Arabidopsis plants. (**B**) Western blot analysis of the *GhDIRP1* protein level in the selected transgenic Arabidopsis plants. (**C**) Comparison of disease phenotype in *GhDIRP1* transgenic Arabidopsis plants after *V. dahliae* infection. (**D**) Comparison of leaf phenotype of different Arabidopsis plants. (**E**) DAB staining results of different Arabidopsis plants upon *V. dahliae* infection. * and ** indicate statistically significance at *p* < 0.05 and *p* < 0.01, respectively.

We further overexpressed *GhDIRP1* in Arabidopsis, and the two transgenics (OE-4 and OE-8) with the highest expression of *GhDIRP1* (Figure 3A,B) were used to study the gene function in response to *V. dahliae* infection.

Wild-type control (WT), transgenic control (GFP), T-DNA insertion mutant (*Atrduf1*), and the two overexpression lines (OE-4 and OE-8) were cultured separately and inoculated with Vd991. Phenotypic observations were made 14 days after inoculation. The results showed that, compared to the wild-type and transgenic control lines (GFP), OE-4 and OE-8 exhibited more pronounced and severe disease symptoms, including increased yellowing and wilting (Figure 3C,D) and stronger DAB staining in leaves (Figure 3E), indicating a severe outbreak of free radicals. In contrast, *Atrduf1*, the line with mutation in the Arabidopsis homolog of *GhDIRP1*, showed fewer disease symptoms, with some individuals being uninfected and no DAB staining in leaves (Figure 3C,E).

The immune response of these lines was further evaluated using several other parameters, including accumulation of pathogen, disease index, plant biomass, and lignin content. Compared to the controls (WT and GFP), OE-4 and OE-8 accumulated significantly more fungal biomass in the roots (Supplementary Figure S3A) and had over 90% of the plants showing severe disease symptoms (grade 3 and 4) (Supplementary Figure S3B). The *Atrduf1* mutant had the lowest accumulation of pathogen and diseased plants (Figure 3A,B). Compared to their corresponding mock-infected plants, the infected plants of all lines had a decreased biomass after infection with Vd991. Among the infected lines, the biomass of OE-4 and OE-8 was significantly lower than that of the controls (WT and GFP), while the biomass of the *Atrduf1* mutant plants was significantly higher (Supplementary Figure S3C). *V. dahliae* infection increased the lignin content in all lines, but the increase was the lowest in OE-4 and OE-8 and the highest in the *Atrduf1* mutant (Supplementary Figure S3D).

In addition, after infection with Vd991, the activity or content of ROS-related enzymes and substances increased in all lines compared to the uninfected lines. OE-4 and OE-8 had a >100% increase in H_2_O_2_ and the lowest increase in the NO content and the POD and SOD activity. By contrast, the *Atrduf1* mutant showed the lowest increase in H_2_O_2_ and the highest increase in the NO content as well as the POD and SOD activity (Supplementary Figure S4A–D).

Together, these results demonstrated that heterogeneously overexpressing *GhDIRP1* in Arabidopsis compromised its disease resistance, and like *GhDIRP1*, *AtRDUF1* is a negative regulator of resistance to *V. dahliae.*

### 2.6. Cloning and Sequence Characterization of GhDIRP1 and Its Homologs from Other Plant Species

**Figure 4:**
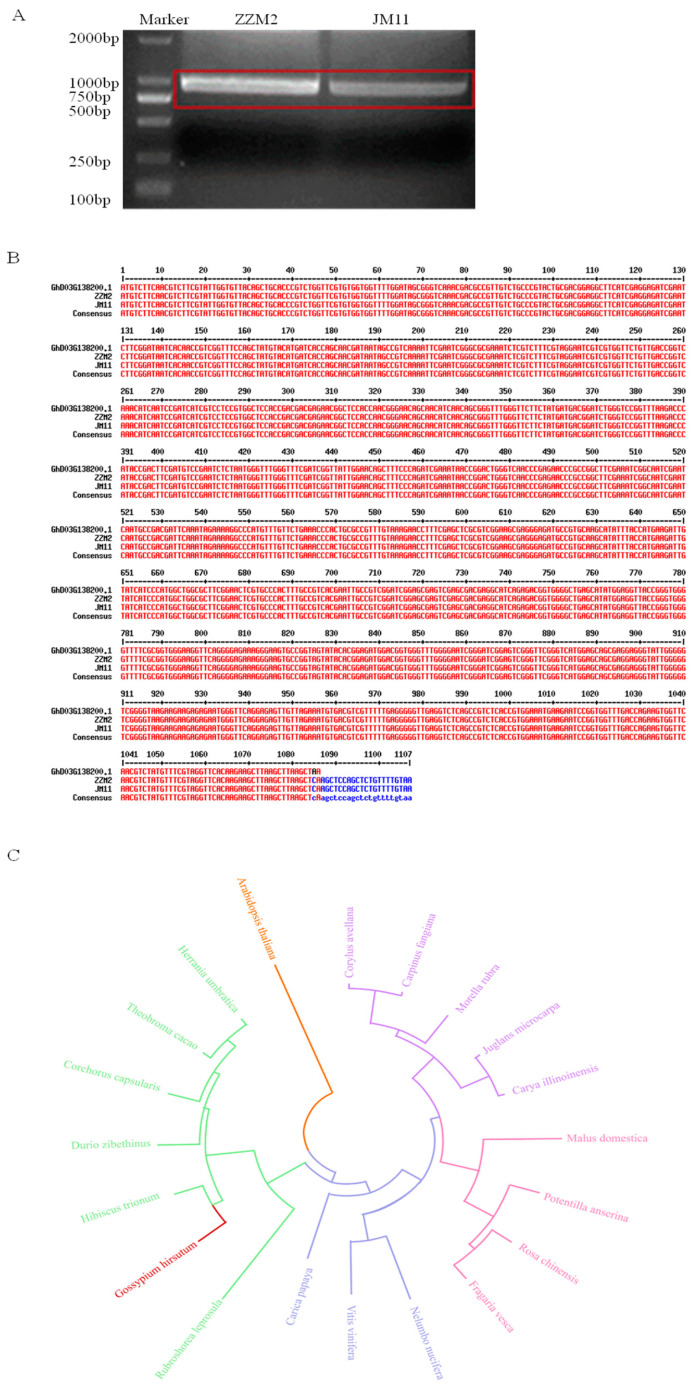
Characterization of *GhDIRP1* sequence. (**A**) Cloning of *GhDIRP1*. The fragments in red box indicate the amplified products of *GhDIRP1* cDNA. (**B**) Comparison of the coding sequences of *GhDIRP1* from ZZM2 and JM11. *Gh_D03G138200* was based on the annotation of the TM-1 reference genome (Sequencing version: G.hirsutum_Genome_HAU_V1.0). (**C**) Phylogenetic tree of GhDIRP1 and its homologous proteins from other plant species. The phylogenetic tree was constructed using MEGA10.1 software by the NJ method.

*Gh_D03G138200* (*GhDIRP1*) was cloned from the ZZM2 and the JM11 (Figure 4A). A sequence comparison showed that there is no difference between the cDNA sequences from ZZM2 and JM11 (Figure 4B). We retrieved the amino acid sequences of GhDIRP1 homologs of several plant species from the NCBI database and compared their matching degree with GhDIRP1. The similarity between GhDIRP1 and its *Arabidopsis thaliana* homolog was only 48.27%, while the similarity between GhDIRP1 and its homologs from the closely related species in the Malvaceae family, such as *Theobroma cacao*, *Hibiscus trionum*, *Durio zibethinus*, *Columbia mallow*, and *Corchorus capsularis*, exceeded 80% (Figure 4C). From the phylogenetic tree, GhDIRP1 clustered together with its homologs from *Theobroma cacao* and *Columbia mallow*, consistent with the evolutionary relationship of the species.

### 2.7. The Promoter of GhDIRP1 from ZZM2 Has a Stronger Activity than That from JM11

**Figure 5:**
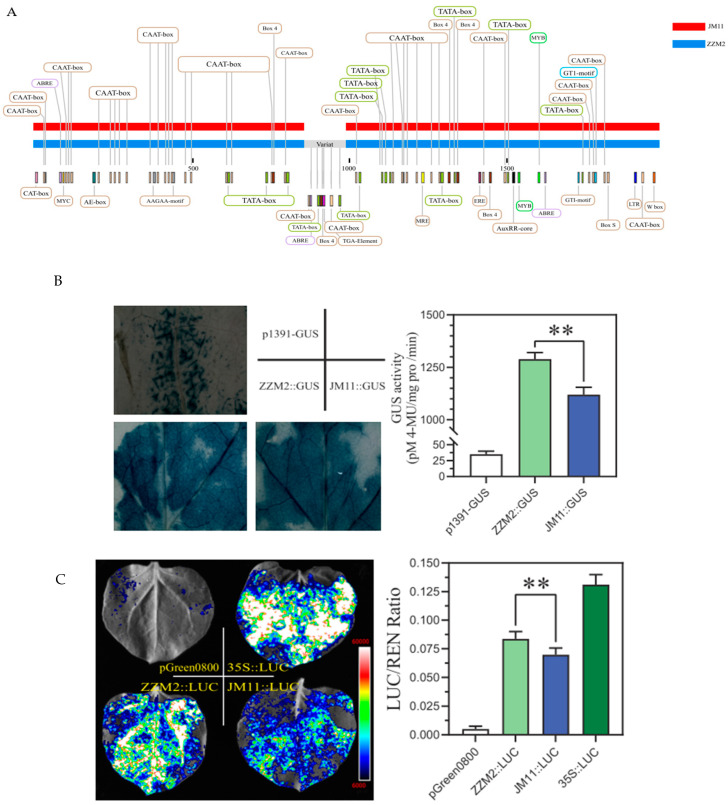
Comparison of the *GhDIRP1* promoter from ZZM2 and JM11. (**A**) Promoter sequence from ZZM2 and JM11. (**B**) Visualization of the promoter activity based on GUS staining (**left**) and quantification of GUS activity (**right**). (**C**) Visualization of the promoter activity based on LUC fluorescence analysis (**left**) and quantification of LUC activity (**right**). **: indicates statistically significant at *p* < 0.01.

Given the identical coding sequence of *GhDIRP1* from the varieties resistant or susceptible to *V. dahliae*, we compared the sequence and activity of the *GhDIRP1* promoter from the two varieties. Compared with the promoter from JM11, the promoter from ZZM2 has a 150 bp insertion at the 1000 bp position, which contains several hormone-responsive elements (Figure 5A). The activity of the promoters from ZZM2 and JM11 was investigated by GUS and LUC reporter vectors. For the GUS reporters, while no obvious difference was observed between the two promoters based on visual inspection (Figure 5B), based on quantification of the GUS enzyme activity, significantly higher activity was observed in the ZZM2 promoter (Figure 5B). For the LUC reporters, a stronger fluorescence signal was observed in the ZZM2 promoter than in the JM11 promoter (Figure 5C), although both were weaker than the control 35S promoter. This observation was further confirmed by quantification of the LUC activity (Figure 5C). Collectively, these results indicated a stronger activity of the promoter from ZZM2 than that from JM11.

### 2.8. GhDIRP1 Is a Membrane Protein

**Figure 6:**
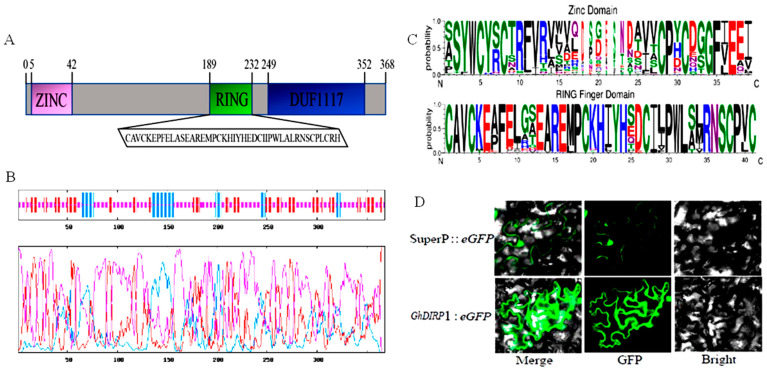
Property and localization of GhDIRP1. (**A**) Analysis of the conserved domains of GhDIRP1. (**B**) Prediction of the secondary structure of GhDIRP1. (**C**) Conservation of the Zinc and RING Finger domains of GhDIRP1. (**D**) Subcellular localization of GhDIRP1.

*GhDIRP1* has an open reading frame (ORF) of 1107 bp, encoding 368 amino acids. The molecular weight of GhDIRP1 predicted by the online tool Prot Param (http://web.expasy.org/protpara, accessed on 12 March 2021) is 40.4 kDa, with a theoretical pI of 6.72. GhDIRP1 possesses three domains: ZINC, RING Finger, and DUF1117 (Figure 6A). The GhDIRP1 protein contains a high proportion (58.70%) of irregular coils, followed by β-extended chains at 27.99% and α-helices at 13.32% (Figure 6B). The RING finger domain with 40–60 amino acids is a special type of zinc finger that binds to a pair of zinc atoms and participates in mediating protein–protein interactions. Like its homologs, GhDIRP1 has the characteristic amino acid pattern of CX2CXnHXnHX2CHnCX2CX2H (X represents any amino acids) in the RING finger domain (Figure 6C). Among the homologs, the degree of variation within this region is very low, indicating high conservation of the RING domain. To know the subcellular localization of GhDIRP1, it was fused with GFP and expressed in tobacco leaf epidermal cells. The results indicated that GhDIRP1 is localized at the cell membrane (Figure 6D).

### 2.9. GhDIRP1 Is a Functional Ubiquitin Ligase

**Figure 7:**
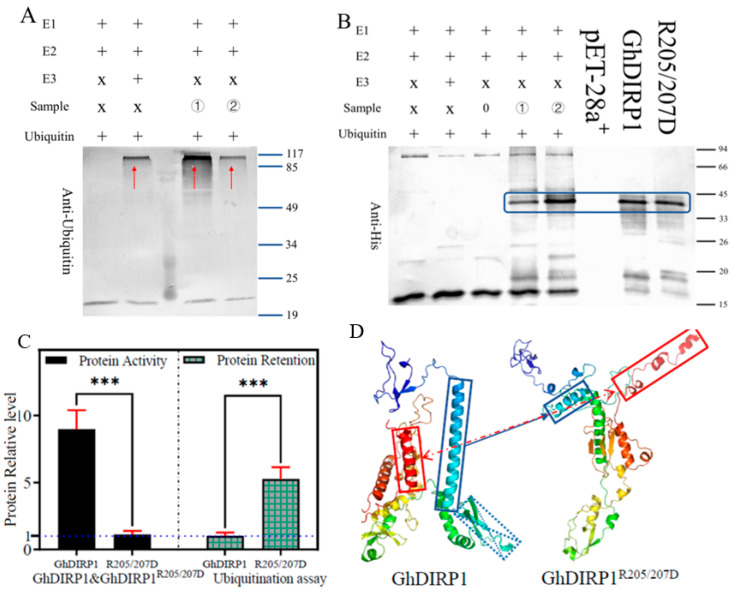
GhDIRP1 is a functional ubiquitin ligase. (**A**) In vitro ubiquitination activity detection (anti-Ubs). ① and ② indicate the presence of GhDIRP1 and GhDIRP1^R205/207D^ proteins, respectively. Red arrows indicate the results immunoblotted with anti-Ub antibody. (**B**) Substrate residue detection (anti-His). ① and ② indicate the presence of GhDIRP1 and GhDIRP1^R205/207D^ proteins, respectively. Blue box indicates the results immunoblotted with anti-His antibody. (**C**) Quantification of in vitro ubiquitination activity based on protein activity and protein retention. *: indicates statistical difference (***: *p* < 0.001). (**D**) The effect of the mutations in the RING domain on the structure of GhDIRP1.

Next, we tested the E3 ubiquitin ligase activity of GhDIRP1 in vitro using a commercial kit. The results showed ubiquitin monomers forming chains and producing a diffuse smear in the lanes (Figure 7A), indicating that GhDIRP1 is a functional E3 ubiquitin ligase. To further test the importance of the RING domain in the ubiquitination activity of GhDIRP1, two amino acids in the domain were mutated (positions 205 and 207). As a result, the E3 ligase catalytic activity of GhDIRP1^R205/207D^ was greatly reduced compared to that of GhDIRP1 (Figure 7B). Multiple in vitro ubiquitination experiments were conducted to generate results for statistical quantification of the effect of the mutations in the RING domain on the activity of GhDIRP1 (based on quantifying the grayscale values of each lane using ImageJ). The results showed that the in vitro ubiquitination activity of GhDIRP1 was 7–9-times higher than that of GhDIRP1^R205/207D^, and the protein retention was 4–5-times higher in GhDIRP1^R205/207D^ than in GhDIRP1 (Figure 7C). These results indicate the importance of the RING domain for the E3 ubiquitin ligase function of GhDIRP1. To know the effect of the mutations in the RING domain on the protein structure, we used the Tencent Cloud DeepMind Drug platform (https://drug.ai.tencent.com/console/, accessed on 29 December 2023) to predict the structure of the two proteins (Figure 7D). It was evident that GhDIRP1^R205/207D^ exhibited shifts and isomerization of two α-helices and the loss of a complete β-folded sheet compared to GhDIRP1. This suggests that the mutations at positions 205 and 207 in the amino acid sequence of GhDIRP1 led to structural rearrangement, thereby affecting the in vitro ubiquitination function.

## 3. Discussion

Ubiquitination is a fundamental protein degradation pathway, and E3 ubiquitin ligases have diverse biological functions, including cell differentiation, hormone synthesis, basic metabolism, and disease resistance. The ubiquitin-mediated 26S proteasome system precisely regulates various physiological and biochemical processes in plants by targeting specific substrate proteins for degradation. As an important component of the UPS pathway, E3 ubiquitin ligases play key roles in plant responses to various stimuli [30,31]. For example, the cotton E3 ubiquitin ligase *GhPUB17* negatively regulates resistance to *V. dahliae*, and the antifungal protein *GhCyP3* interacts with *GhPUB17*, inhibiting its ligase activity and playing a critical role in the cotton–Verticillium interaction [15,16,17]. In this study, we demonstrated that the *GhDIRP1* protein has a strong ubiquitin ligase activity, and the RING domain is crucial for the role of *GhDIRP1* as a ubiquitin ligase, because point mutations (positions 205 and 207) in the RING domain greatly reduce the in vitro ubiquitination ability of *GhDIRP1*, indicating that the RING domain is necessary for *GhDIRP1* to exercise its ubiquitin ligase activity. The reduced ubiquitination activity of GhDIRP1^R205/207D^ is likely due to the changed protein structure caused by the point mutations. De novo protein structure prediction showed that GhDIRP1^R205/207D^ had a looser structure than GhDIRP1, which might be closely related to the difference in ubiquitination activity between them. It was reported that plants with altered RING-type E3 ubiquitin ligase gene expression levels (e.g., overexpression or silencing) exhibit modulated defense responses following pathogen infection [30]. In this study, we functionally characterized *GhDIRP1* in resistance against the fungal pathogen *V. dahliae*. *GhDIRP1* was differentially expressed between cotton varieties resistant (ZZM2) or susceptible (JM11) to *V. dahliae*, likely due to the sequence difference in their promoters because ZZM2 and JM11 had identical coding sequence. The main difference in the *GhDIRP1* promoter from ZZM2 and JM11 was a 150 bp insertion in ZZM2, which includes several hormone response elements. In vitro GUS and LUC assays indicated that the activity of the *GhDIRP1* promoter from ZZM2 was 20–30% higher than that from JM11. Subcellular localization results showed that GhDIRP1 is membrane-associated, suggesting its potential role in protein degradation at the cell membrane.

RING-containing proteins have been widely reported to play a substantial role in plant disease resistance [32]. The Arabidopsis RING zinc finger protein ATL9, induced by chitin, is an E3 ubiquitin ligase that plays a role in basal resistance to the biotrophic fungus *Golovinomyces cichoracearum* [33]. The rice RING-type Ubiquitin Ligase protein APIP10 negatively regulates basal defense by interacting with two rice transcription factors and promoting their degradation through the 26S proteasome pathway [19,20].

Among the RING-containing proteins identified, AtRDUF1 and AtRDUF2, two RDUF proteins containing both RING domain and unknown function (DUF) 1117 domain, respond to chitin, a plant defense elicitor, with 7.9- and 9.0-fold increases in gene expression 30 min after induction, respectively [34], implying the role of RING-RDUF1117 in biotic stress. In this study, we characterized GhDIRP1 and found it to possess both RING finger and DUF1117 domains, implying that GhDIRP1 belongs to RDUF proteins. What’s more, we demonstrated that *GhDIRP1* is a negative regulator of *V. dahliae* resistance by silencing *GhDIRP1* in cotton and heterogeneously overexpressing the gene in Arabidopsis. The current results are not consistent with a previous study indicating that Arabidopsis plants that overexpress *GhRDUF4D* were more resistant to *V. dahliae*, and that *GhRDUF4D* down-regulation in cotton plants made them more sensitive to *V. dahliae* infection, compared with the control [35]. However, the current results demonstrated the importance of RDUF protein in *V. dahliae* resistance.

Hormones play a vital role in plant resistance to microorganisms, including pathogenic fungi. JA, SA, and ET are the primary hormones involved in plant immunity [36,37,38]. In this study, many cis-elements involved in hormones were detected in the *GhDIRP1* promoter regions (Figure 3A), and the expression level of *GhDIRP1* in ZZM2 was induced by ethylene and salicylate but repressed by jasmonate (Figure 5B), suggesting that *GhDIRP1* may be involved in the signaling pathways of all these hormones. What’s more, silencing *GhDIRP1* induces expression changes in the marker genes of the JA and SA signaling pathways, which might contribute to explain that silencing *GhDIRP1* in cotton by VIGS-enhanced resistance to *V. dahliae*. However, the immune response mediated by *GhDIRP1* involves multiple hormone-signaling pathways, the lignin biosynthesis pathway, and ROS signaling. How these pathways are regulated by *GhDIRP1* relies on identification of the direct targets of GhDIRP1.

Many U-box E3 ubiquitin ligases are involved in stress responses, including TaPUB1 in salt stress [39], CaPUB1 in cold and drought stress [40], and AtPUB48 in heat stress [31,41]. The cotton U-box E3 ubiquitin ligase GhPUB17 negatively regulates resistance to *V. dahliae*, and the antifungal protein GhCyP3 interacts with GhPUB17, inhibiting its ligase activity and playing a critical role in the cotton–Verticillium interaction [15,16,17].

The RING E3 ligase KEEP ON GOING (KEG) negatively regulates the ABA signaling pathway [42,43] and is an important factor in the jasmonic acid (JA) signaling pathway [44]. In Arabidopsis, a new E3 ubiquitin ligase, PUB4, which interacts with CERK-1, is involved in the regulation of MAMP-triggered immune responses [45]. The Arabidopsis RING zinc finger protein ATL9, induced by chitin, is an E3 ubiquitin ligase that plays a role in basal resistance to the biotrophic fungus *G. cichoracearum*. ATL9 contains two transmembrane domains, a RING zinc finger domain, and a PEST domain. It was found that the evolutionarily conserved Arabidopsis E3 ligase *AtCHIP* positively regulates low-temperature resistance in plants [46]. The RING-H2 type E3 ligase OsSIRH214 regulates salt tolerance in rice by ubiquitinating the salt-related protein OsHKT2 and enhancing salt tolerance [47].

In cotton, an E3 ubiquitin ligase-encoding gene RDUF (RING-DUF1117) has been previously identified, and expression analysis showed that *GhRDUF* was widely expressed under cotton growth and abiotic stress [35]. Many cis-elements associated with hormonal responses and environmental stress factors are found in the *GhRDUF* promoters. *GhRDUF* responds to cold, drought and salt stress, and is sensitive to jasmonic acid, salicylic acid, and ethylene signal. At the same time, the expression level of *GhRDUF4D* was enhanced after the infection of *V. dahliae*. Overexpressing *GhRDUF4D* in *Arabidopsis thaliana* plants enhances resistance to *V. dahliae*, and silencing the gene in cotton plants reduces resistance to *V. dahlia* [35]. In this study, we proved that GhDIRP1, as an RDUF protein, regulates *V. dahliae* resistance by modulating the hormone signal, implying the roles of RDUF protein in responding to stress factors.

Silencing *GhDIRP1* in cotton by VIGS enhanced resistance to *V. dahliae*, while overexpressing *GhDIRP1* in Arabidopsis compromised resistance, implying that *GhDIRP1* is a negative regulator of resistance against *V. dahliae*. The immune response mediated by *GhDIRP1* involves multiple hormone-signaling pathways, the lignin biosynthesis pathway, and ROS signaling. How these pathways are regulated by *GhDIRP1* relies on identification of the direct targets of *GhDIRP1*.

## 4. Materials and Methods

### 4.1. Plant Materials, Fungal Strain, and Growth Conditions

The seeds of cotton varieties Zhongzhimian 2 (ZZM2; resistant to *V. dahliae*) and Jimian 11 (JM11; susceptible to *V. dahliae*) were kindly provided by professor Heqin Zhu from the Institute of Cotton Research of Chinese Academy of Agricultural Sciences. Cotton seeds were sown in a 1:1 mixture of nutrient soil and vermiculite and grown in a greenhouse (16 h of light/8 h of darkness, 23 °C, 65% humidity). *Nicotiana benthamiana* and *A. thaliana* (Columbia ecotype) were grown in eutrophic soil at 23 °C, 65% humidity, 16 h of light/8 h of dark cycling.

A highly pathogenic strain of defoliation-inducing fungus *V. dahliae*, Vd991, was provided by the Cotton Research Institute of the Chinese Academy of Agricultural Sciences. The strain was cultured at 25 °C on potato dextrose agar (PDA) plates for 7–10 days or in potato dextrose broth (PDB) on a shaker at 200 rpm and 25 °C for 2–3 days.

### 4.2. Analysis of Verticillium Wilt and Hormone-Induced Gene Expression

When the third true leaf unfolded, cotton seedlings were treated with *V. dahliae*, salicylic acid (SA), methyl jasmonate (JA), abscisic acid (ABA), and ethylene (ETH) (Solarbio, Beijing, China). *V. dahliae* infection was carried out by dipping the root in 10 mL of conidial suspensions for 5 min with a spore concentration of 1 × 10^7^ conidia/mL. Roots were harvested at time intervals of 0, 6, 12, 24, 36, 48, 72, and 96 h after infection and stored at −80 °C. The experiment was conducted with three biological replicates. Hormone treatment was applied to cotton seedlings in the three-leaf stage by spraying 200 µM JA, 200 µM ETH, or 2 mM SA, with an equal amount of double-distilled water as the control. Leaves were collected at 0, 6, 12, 24, and 48 h after processing and stored at −80 °C.

### 4.3. Gene Silencing and Pathogen Inoculation

The pTRV2 vector from our lab was digested with *Eco*RI and *Bam*HI (New England Biolabs, Shanghai, China) restriction enzymes. The target fragment (1107 bp) was ligated to the tobacco rattle virus (TRV) vector using the seamless cloning approach (Vazyme, Nanjing, China). All vectors (pTRV2::00, pTRV2::GhDIRP1, and the positive control vector TRV-PDS) were transformed into *Agrobacterium tumefaciens* strain GV3101 through freeze–thaw transformation. Eleven-day-old cotton seedlings were injected with equal amounts of the TRV vectors. After incubation in the dark for 24 h, the cotton seedlings were transferred to a greenhouse (16 h of light/8 h of darkness, 23 °C, 65% humidity). When the leaves of the positive control plants treated with TRV-PDS began to fade and turned white (about 10 days after injection), cotton leaf samples were infiltrated with pTRV2::00 and pTRV2:GhDIRP1 and collected in triplicate. Total RNA was extracted using RNAprep Pure plant kit (TIANGEN, Beijing, China) and converted into cDNA for qRT-PCR using ChamQ SYBR qPCR Master Mix (Vazyme, Nanjing, China). The silencing efficiency of the target gene was detected according to results of qRT-PCR. After confirming the silencing of the target gene, the cotton plants were inoculated with Vd991, as mentioned.

### 4.4. Detection of Necrotic Cells and Accumulation of Reactive Oxygen Species (ROS)

Seven days after inoculation with Vd991, cotton seedlings were harvested and boiled for 2 min. Then, leaves were soaked in a solution of trypan blue dye (1 g phenol, 1 mg trypan blue,1 mL lactic acid, 1 mL glycerol diluted in 1 mL sterile distilled water) overnight at room temperature (Solarbio, Beijing, China). Subsequently, the samples were immersed in a 1.25 kg/L hypochlorite solution for 3 days. Then, leaves were examined using a stereo microscope, and photos were taken. The accumulation of reactive oxygen species in cotton leaves was observed using 3,3′-diaminobenzidine (DAB) staining. For this purpose, two true leaves were randomly selected and soaked in a DAB staining solution (DAB concentration: one thousandth, pH = 3.0). After vacuum infiltration, they were incubated overnight at room temperature. Ethanol was used to destain the leaves until they turned completely chlorosis, and observations were made under a stereomicroscope, with photographs taken and recorded.

### 4.5. Measurement of NO, H_2_O_2_, POD, and SOD Activity

The abundance of immune response-related chemicals, including SOD, POD, H_2_O_2_, NO, and total protein, was measured. The first true leaf of cotton seedlings was ground into power in liquid nitrogen using a mortar and pestle and homogenized in 50 mM phosphate buffer (pH 7.0). After centrifugation at 13,000 *g* for 20 min, SOD, POD, H_2_O_2_, and NO (Solarbio, Beijing, China) were detected using commercially available assay kits according to the manufacturer’s instructions. The total protein concentration was measured in the supernatant using the BCA protein assay kit.

### 4.6. Transformation of Arabidopsis

In order to construct an overexpression vector, we amplified the ORF of *GhDIRP1* and inserted it into the P2300-Ov vector through fusion cloning. The overexpression vector was introduced into *A. tumefaciens* strain GV3101, which was used to transform Arabidopsis. Agrobacterium-mediated transformation was performed using the floral dip method with Arabidopsis Col-0 plants. Transgenic lines were screened using the TransDirect Plant Tissue PCR Kit and qPCR to detect transgene insertion and optimal segregation ratios. For subsequent functional studies, stable homozygous T3 lines with high *GhDIRP1* expression were selected.

### 4.7. Gene Cloning

The gene-specific primers were designed based on the coding sequence of *Gh_D03G138200*. The entire ORF of *GhDIRP1* was amplified from cDNA of ZZM2 and JM11. The cDNA was amplified by high-fidelity enzyme KOD-Plus-Neo (KOD-401, Toyobo, Japan), and PCR products were purified by 1% agarose gel electrophoresis. The purified fragments were connected to the T-vector using the 5 min TA/Blunt-Zero cloning kit (C601, Vazyme, Nanjing, China), and the recombinant products were transformed into *Escherichia coli* DH5α strain. We then randomly selected at least 5 clones and sequenced the ORF of ZZM2 or JM11.

### 4.8. Analysis of GhDIRP1 Sequence and Its Subcellular Localization

The amino acid sequence of GhDIRP1 and its homologous proteins from Arabidopsis, Hibiscus, Durian, Hemp, and Cocoa were downloaded from the NCBI database (https://www.ncbi.nlm.nih.gov/). A neighbor-joining (NJ) method was employed to construct the phylogenetic tree using MEGA software. Amino acid sequence alignment of GhDIRP1 was performed using SnapGene 3.2.1 software. Protein structure prediction and analysis were carried out using the online analysis software SOPMA (http://expasy.org/tools/sopma, accessed on 15 June 2024), and subcellular localization prediction was conducted using UniProt (https://www.uniprot.org/).

The ORF region of *GhDIRP1* was amplified and cloned into the p1300-Super-GFP vector to generate a fusion protein with GFP at the C-terminus of GhDIRP1. The p1300-Super-GFP vector was served as a control in the analysis of subcellular localization. The fungal suspensions were injected into *Agrobacterium tumefaciens* strain GV3101, and the bacterial suspensions (OD600 = 0.5~0.8) were injected into tobacco leaves. After 2 days of incubation, the leaves were cut, and GFP fluorescence characteristics were observed under a confocal microscope (excitation wavelength: 488 nm, emission wavelength: 505~530 nm).

### 4.9. Promoter Amplification, GUS, and LUC Reporter Experiments

Although the CDS sequences of *GhDIRP1* cloned from ZZM2 and JM11 were identical, the expression trend of *GhDIRP1* was completely opposite in these two varieties after inoculation with *V. dahliae*. Therefore, the 2000 bp promoter was amplified from the two varieties using specific primers to analyze their activity by fusing each of them with GUS (β-glucuronidase) or LUC (Luciferase). The promoter::GUS vectors were transformed into *Agrobacterium* GV3301. After culturing, the bacteria were collected and re-suspended, the OD600 of the suspension was adjusted to 1.0, and the culture was used to inoculate tobacco leaves by injection. GUS activity was measured 2 days after inoculation. The promoter::LUC vectors were constructed and used to inoculate tobacco leaves in the same way as the promoter::GUS vectors, and D-fluorescein sodium salt was sprayed and exposed to the fluorescence imager 2 days after inoculation.

### 4.10. In Vitro Ubiquitination Assay

The entire *GhDIRP1* sequence was cloned into the pET28a+ vector and used for transformation into *E. coli* BL21. The GhDIRP1: His-tagged protein was purified from *E. coli* BL21(DE3) pLysS using His-tag protein purification resin (Beyotime, Shanghai, China). Ubiquitination reactions were performed using the ubiquitin-activating enzyme solution (E1), Hdm2 (E2), and E3 ligase buffer from the E3 enzyme auto-ubiquitination assay kit (Abcam, Cambridge, UK). The reaction mixture was incubated at 37 °C for 2 h and then separated on a 12% SDS-PAGE gel. To detect ubiquitination, the separated reaction products were transferred onto a PVDF membrane and detected using anti ubiquitin antibodies and anti-rabbit IgG horseradish peroxidase secondary antibodies.

### 4.11. Statistical Analysis

All experiments were conducted at least three times. Statistical analysis was performed using Graph Pad Prism^®^ 6 software (Graph Pad, San Diego, CA, USA). Analysis of variance (ANOVA) was conducted, followed by Dunn’s multiple comparison test. A *p*-value of ≤0.05 was considered statistically significant.

## 5. Conclusions

In conclusion, we demonstrated that *Gh_D03G138200* or *GhDIRP1* negatively regulates the immune response against *V. dahliae* via multiple pathways, including hormone signaling, reactive oxygen species, and lignin biosynthesis. GhDIRP1 is an RDUF protein and possesses ubiquitination activity. While the direct targets of GhDIRP1 are yet to be identified, the results of this study link ubiquitination to immune response in cotton and provide a candidate gene for cotton breeding for Verticillium wilt resistance.

## Data Availability

Data are contained within the article and Supplementary Materials.

## Supplementary Materials

The following supporting information can be downloaded at: https://www.mdpi.com/article/10.3390/plants13152047/s1, Figure S1. Determination of ROS-related enzymes. Figure S2. Analysis of disease resistance-related genes by qRT-PCR in *GhDIRP1*-silenced and -unsilenced ZZM2 and JM11 plants upon *Verticillium dahliae* infection. Figure S3. Comparison of the pathogen content, the level of disease, biomass, and lignin content in transgenic Arabidopsis plants after *Verticillium dahliae* infection. Figure S4. Determination of ROS-related enzyme activities in transgenic Arabidopsis.
