# Supplementary material for: The RING-Type E3 Ubiquitin Ligase Gene GhDIRP1 Negatively Regulates Verticillium dahliae Resistance in Cotton (Gossypium hirsutum)
Source: Plants (Basel). 2024 Jul 25;13(15):2047. doi: 10.3390/plants13152047 (PMC11314081; doi:10.3390/plants13152047)

Supplementary Figure S1. Determination of ROS-related enzymes. (A) Chlorophyll content. (B) Protein content. (C) H_2O_2 content. (D) SOD content. (E) NO content. (F) POD content. ns: no statistical difference; * and **: statistical difference at $p<0.05$ and $p<0.01$, respectively.

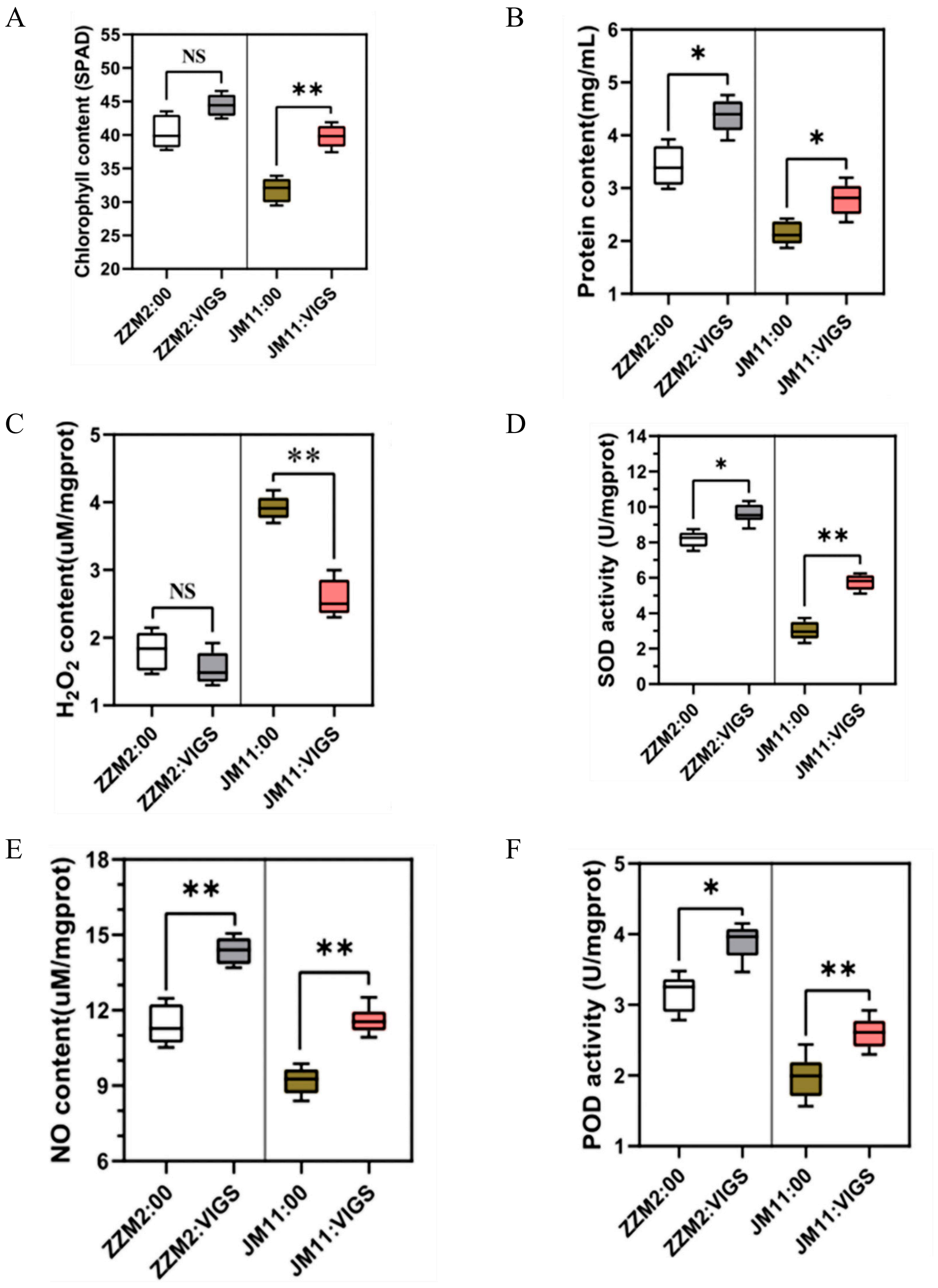

Supplementary Figure S2. Analysis of disease resistance-related genes by qRT-PCR in *GhDIRP1*-silenced and -unsilenced ZZM2 and JM11 plants upon *Verticillium dahliae* infection. (A) Changes of disease resistance-related genes in *GhDIRP1*-silenced and -unsilenced ZZM2 plants. (B) Changes of disease resistance-related genes in *GhDIRP1*-silenced and -unsilenced JM11 plants. ns: no statistical difference; * and **: statistical difference at p<0.05 and p<0.01, respectively.

A

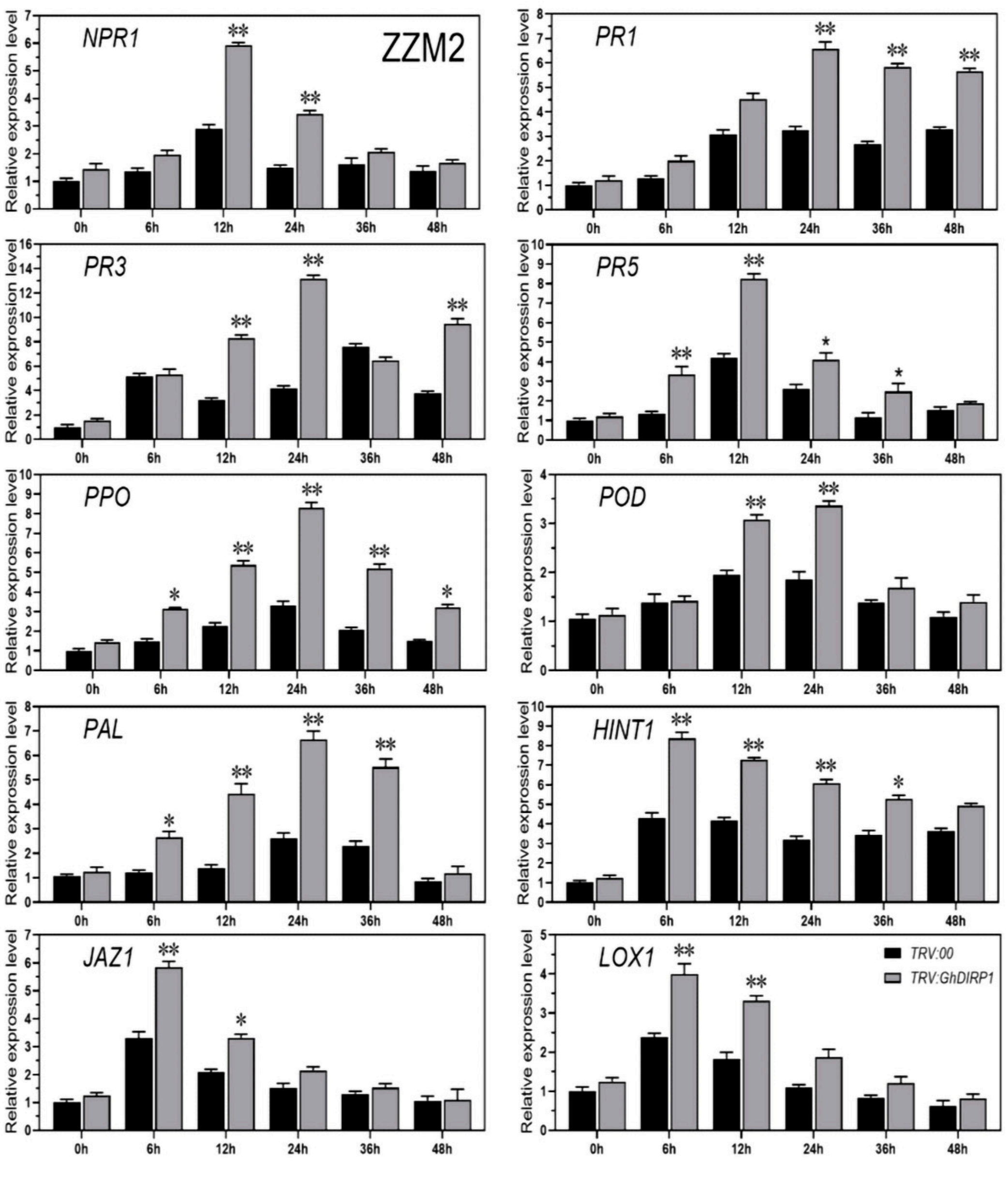

B

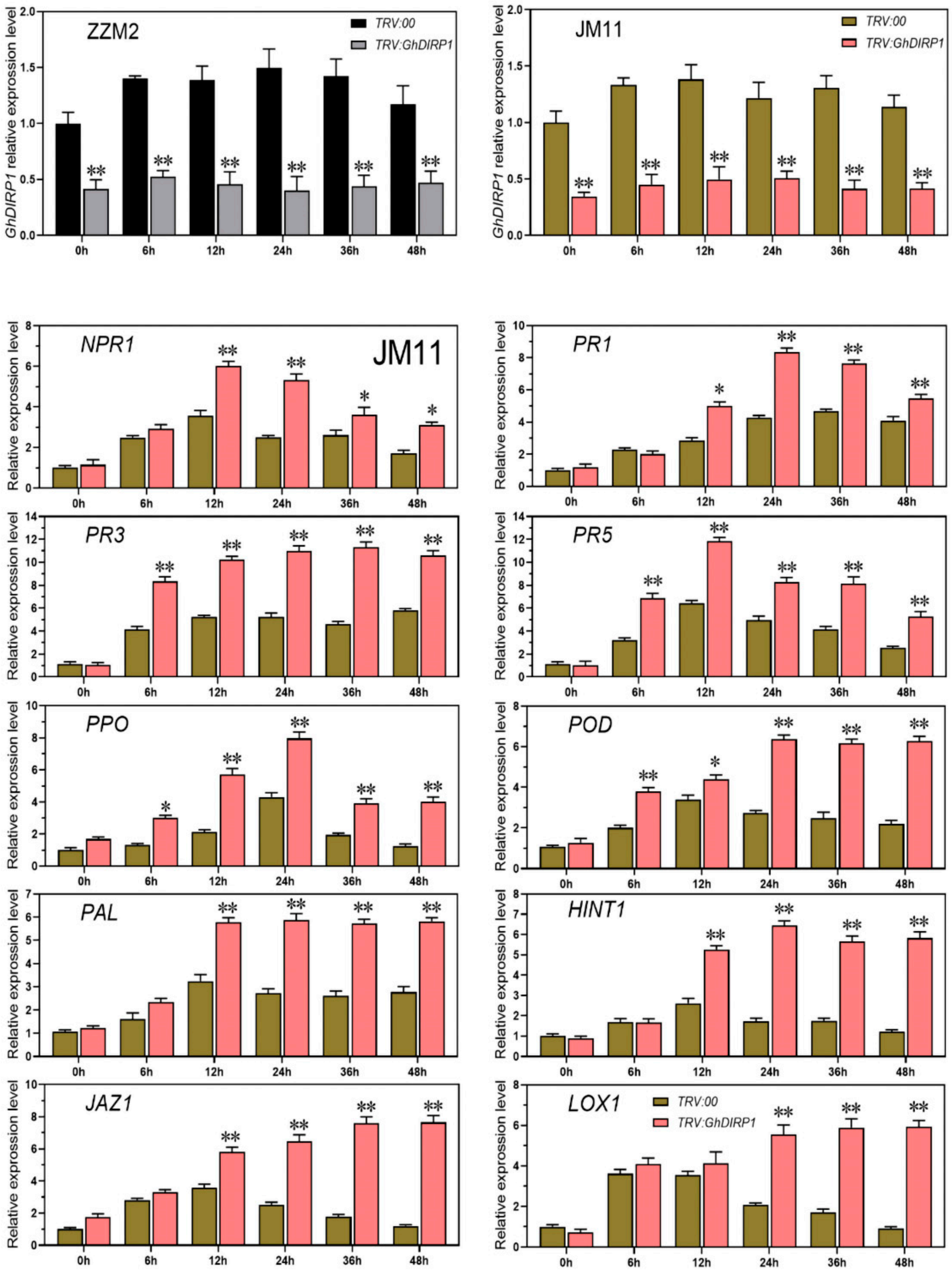

ZZM2
TRV:00
TRV:GhDIRP1
GhDIRP1 relative expression level
JM11
NPR1
PR1
PR3
PR5
PPO
POD
PAL
HINT1
JAZ1
LOX1
Relative expression level
0h
6h
12h
24h
36h
48h

Supplementary Figure S3. Comparison of the pathogen content, the level of disease, biomass, and lignin content in transgenic Arabidopsis plants after *Verticillium dahliae* infection. (A) The pathogen content in roots. (B) Disease index. (C) Comparison of biomass between the mock and *Verticillium dahliae* infected plants. (D) Comparison of lignin content between the mock and *Verticillium dahliae* infected plants. ns: no statistical difference; * and **: statistical difference at p<0.05 and p<0.01, respectively.

A

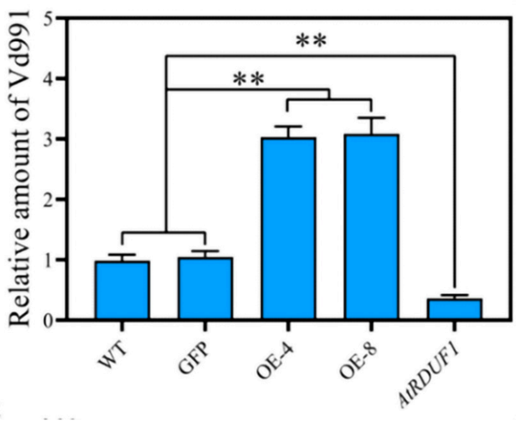

B

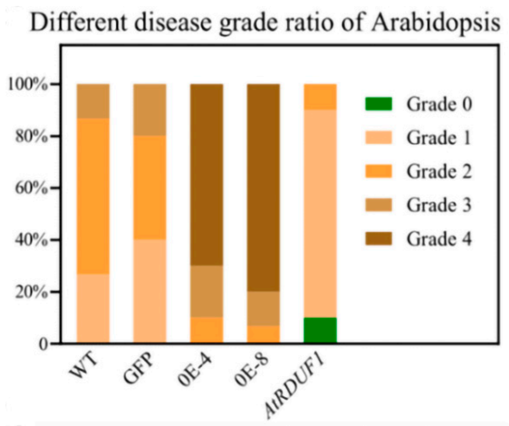

C

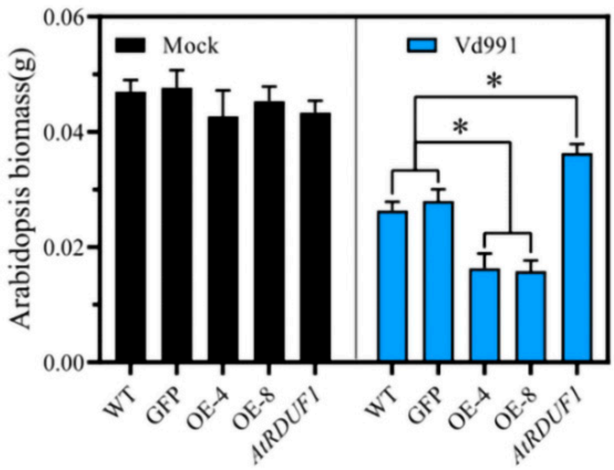

D

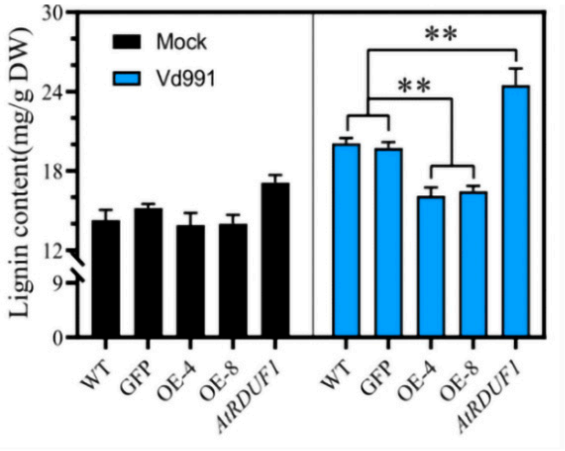

Supplementary Figure S4. Determination of ROS-related enzyme activities in transgenic Arabidopsis. (A) H_2O_2 content. (B) NO content. (C) POD content. (D) SOD content. ** indicates statistical difference at $p<0.01$.

A

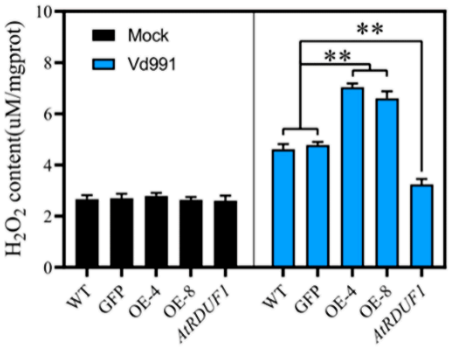

B

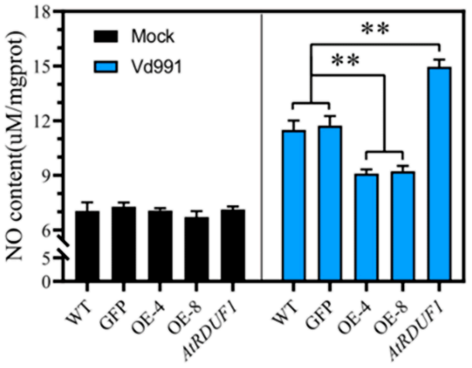

C

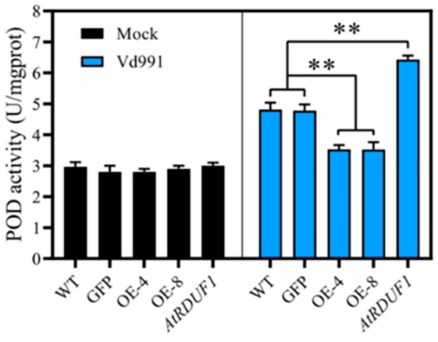

D

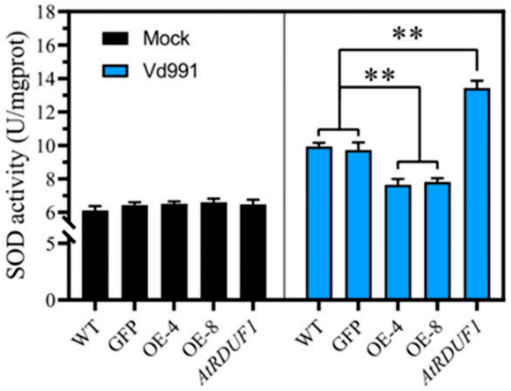

Supplement: Supplementary file 1 [file plants-13-02047-s001.zip › plants-3084448-supplementary.pdf]
